# Experimental study on synergistic pulses improving the tumor microenvironment and assisting immunotherapy

**DOI:** 10.3389/fimmu.2026.1751463

**Published:** 2026-03-11

**Authors:** Shoulong Dong, Zongnan Liu, Zetong Wang, Xinyu Peng, Qinyu Huang, Haobo Yang, Yuan Lei, Chenguo Yao

**Affiliations:** National Key Laboratory of New Technology for Power Transmission and Transformation Equipment, Chongqing University, Chongqing, China

**Keywords:** immunotherapy, NK cells, synergistic pulses, tumor microenvironment, tumor remodeling

## Abstract

The development and metastasis of tumors depend on the highly complex and heterogeneous tumor microenvironment (TME). Synergistic pulsed electric fields refer to a sequential combination of high-voltage nanosecond pulses and low-voltage microsecond pulses. No systematic research has been reported on the ameliorative effect of this field on the TME and the related mechanisms. This study used tumor-bearing models established with male Balb/c nude mice and male C57BL/6 mice as research subjects. At the molecular, cellular, and tissue levels, this study employed Masson staining, immunofluorescence staining, immunohistochemical staining, and *in vivo* small animal imaging technology. This study multi-angle analyzed the effect of synergistic pulse treatment on key physical and chemical indicators of the tumor microenvironment (TME) for the first time. It also explored the effect of its combined application with exogenously injected natural killer (NK) cell therapy. Experimental results showed that synergistic pulse treatment could effectively improve the physical properties of the TME. It reduced tumor stiffness and density, and alleviated the hypoxic state. Meanwhile, it could stimulate the body’s anti-tumor immune response and promote the infiltration of cytotoxic immune cells into tumors. Furthermore, this pulse could significantly promote the enrichment and intratumoral penetration of exogenously injected NK cells at tumors, thereby boosting cellular immunotherapy efficacy. This study demonstrated that synergistic pulsed electric fields can potentially remodel the TME and enhance immune cells’ anti-tumor effect for tumor ablation. It provides a new technical direction for inhibiting tumor recurrence and metastasis and demonstrates potential for clinical translation.

## 1 Introduction

T umor ablation via irreversible electroporation—especially traditional microsecond pulsed electric fields—exhibits unique minimally invasive, non-thermal, and selective advantages in clinical treatment (1). However, traditional microsecond pulsed electric field ablation faces ablation range limitations when treating deep or large-sized tumors, hindering ideal treatment precision. This inadequate precision further leads to suboptimal ablation outcomes, failing to achieve the desired therapeutic effect on the targeted tumor tissue. This, in turn, leads to residual tumor cells and increases the risk of tumor recurrence and metastasis (2, 3). In addition, nanosecond pulsed ablation technology, characterized by intracellular effects, can accurately target organelles (4). However, its effective action range is more concentrated, making the aforementioned limitation of “multiple needles and multiple sessions” more prominent (5). Synergistic pulses—a sequential combination of high-voltage nanosecond pulses and low-voltage microsecond pulses—provide a new approach to overcome this technical bottleneck. Studies have shown this pulse combination achieves simultaneous electroporation of cell and nuclear membranes, significantly expanding tissue ablation range (6). Moreover, its ablation efficacy has been validated in tumor cells (7), normal rabbit liver tissues (8), and mouse tumors (9), holding promise for addressing the limitations of traditional ablation and the challenge of tumor heterogeneity affecting electroporation.

However, tumor ablation not only requires complete clearance of primary lesions but also effective inhibition of subsequent recurrence and metastasis. This process is closely related to the highly complex and heterogeneous tumor microenvironment (TME) where tumors reside. The TME is an immunosuppressive ecosystem. Its dense and stiff extracellular matrix (10), irregular vascular network (11), and hypoxic (12) and acidic (13, 14) biochemical environment form a solid physical and chemical barrier for tumor cells. This barrier severely inhibits the infiltration and function of endogenous immune cells (e.g., T cells, natural killer (NK) cells), leading to immune escape (15). It also hinders the deep delivery and tumor-site accumulation of exogenous therapeutic agents—such as anti-CTLA-4 (16) carried by nanoparticles (NPs)—and injected immune cells (e.g., NK cells (17), chimeric antigen receptor T (CAR-T) cells (18)). This compromises the efficacy of multiple therapies, including cellular immunotherapy (19). Existing studies have shown that irreversible electroporation (IRE) can specifically disrupt tumor blood vessels (20, 21)while preserving extracellular matrix structure (22). It can also induce systemic anti-tumor immunity with long-lasting protective effects (15).Nanosecond pulsed electric fields have also been shown to significantly alter immune cell infiltration and trigger immune responses (23). In recent years, the therapeutic strategy of combining IRE with immunotherapy has gradually matured.

Multiple clinical trials have shown that NK cell-based immunotherapy after IRE ablation exerts a synergistic effect. It significantly reduces the number of circulating tumor cells and improves patients’ immune function (17, 24, 25). However, systematic research on whether synergistic pulses (with superior ablation efficacy) actively remodel TME physicochemical properties is still lacking. It also remains unclear if they can further stimulate the body’s intrinsic anti-tumor immune response. Additionally, the therapeutic efficacy and related mechanisms of synergistic pulses combined with immunotherapy have not been fully elucidated.

This study first uses two types of tumor-bearing mice as models, aiming to investigate the improvement of the tumor microenvironment (TME) by synergistic pulses. First, it explores whether synergistic pulses can improve key physical and chemical indicators of tumors, such as density, stiffness, and hypoxia. Second, it investigates whether this TME improvement can reverse immunosuppression—thereby stimulating the body’s anti-tumor immune response—and promote the infiltration of endogenous cytotoxic immune cells.

Based on this, animal experiments on synergistic pulses assisting exogenously injected natural killer (NK) cell therapy were conducted. These experiments provide experimental basis for subsequent clinical trials of synergistic pulse ablation combined with cellular immunotherapy.

## 2 Materials and methods

### 2.1 Synergistic pulse treatment protocol

#### 2.1.1 Pulsed electric field experimental platform and measurement system

**Figure 1:**
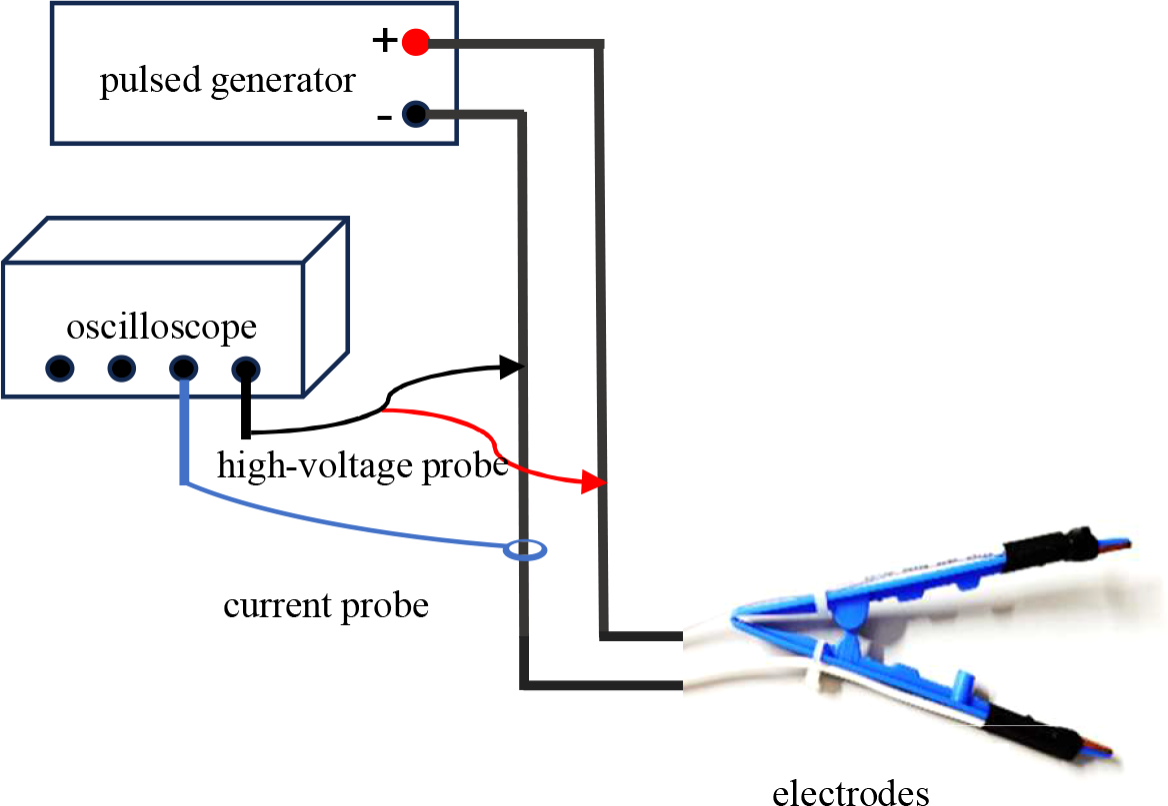
Schematic of the collaborative pulsed electric field and its measurement layout.

During experiments, different electrodes were used based on specific methods, and pulsed electric fields with specific parameters were applied to the load via these electrodes. The experimental wiring diagram is shown in **Figure 1**. The pulse generator used in this study was independently developed by the laboratory. It includes two modules: a nanosecond pulse module and a microsecond pulse module. Via touch buttons on the screen, three output modes can be selected independently: standalone nanosecond pulses, standalone microsecond pulses, and nanosecond-microsecond synergistic pulses. For the nanosecond pulse module: the output voltage amplitude can reach 15 kV, and the pulse width is continuously adjustable from 200 ns to 1 μs. For the microsecond pulse module: the output voltage amplitude can reach 5 kV, and the pulse width is continuously adjustable from 10 μs to 100 μs. The repetition frequency is continuously adjustable from 0.01 Hz to 1 kHz. The nanosecond-microsecond pulse interval is continuously adjustable from 1 μs to 1 ms. By cooperating with an oscilloscope (Wave Pro 760Zi-A, LeCroy, 6 GHz), a PPE 5 kV high-voltage probe (LeCroy, 6 kV, 400 MHz) and a Pearson current sensor (Person 411, 5000 A, 20 MHz) were installed on the lead wires of the pulse generator’s positive and negative electrodes. These devices were used to real-time monitor the voltage waveform across the load and the current waveform flowing through it during the experiment.

#### 2.1.2 Establishment of tumor-bearing mouse models

Ten male Balb/c nude mice (Specific-pathogen-free (SPF) grade, 6–8 weeks old, 15–20 g) and ten male C57BL/6 mice (conventional grade, 6–8 weeks old, 15–20 g) were used as experimental subjects. All mice were housed in an SPF-grade animal facility. Fresh water and bedding were replaced every two days.

Cages, bedding, and water bottles were sterilized via autoclaving. The animal housing and experimental procedures strictly complied with the *Regulations on the Administration of Experimental Animals of the People’s Republic of China*. Approximately 3 mm × 3 mm tumor fragments of the Hepg2 hepatocellular carcinoma cell line were subcutaneously implanted in the dorsal region of each nude mouse. Over the following two weeks, the mice were weighed continuously, and tumor sizes were measured using a vernier caliper to monitor tumor growth. When the tumors grew to approximately 150 mm³, the mice were anesthetized via intraperitoneal injection of 1% sodium pentobarbital. The sodium pentobarbital was prepared with physiological saline at an injection dose of 50 mg/kg. Subsequently, the mice were cleaned and disinfected with alcohol swabs, and ear tags were applied for pre-experiment preparation. The calculation formula for mouse tumor volume is shown in (Equation 1).

(1) $$V\;=\;\frac{L\times W^{2}}{2}$$

Among them, *V*, *L*, *W* and are respectively the tumor volume, tumor length, and tumor width.

#### 2.1.3 Animal experiments

**Figure 2:**
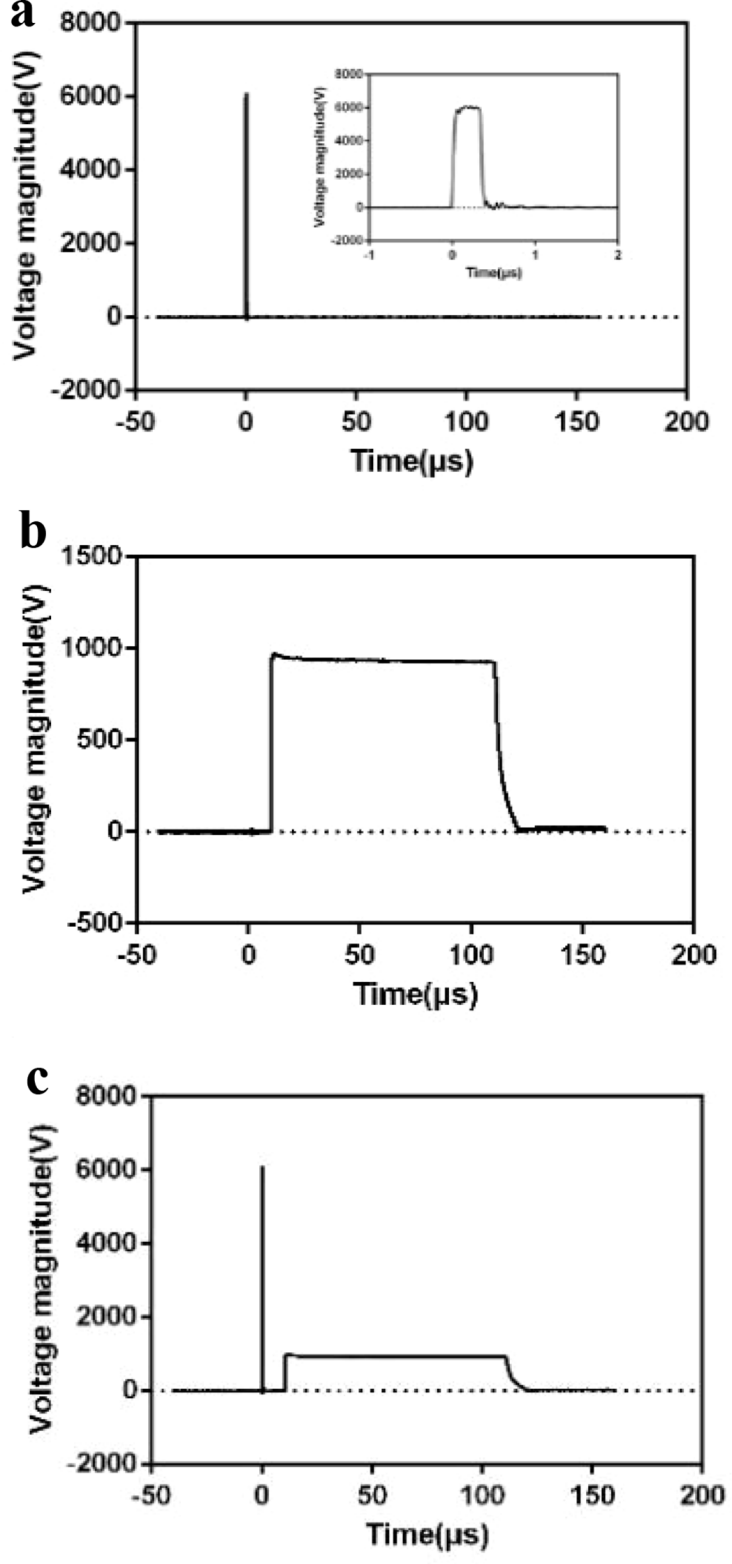
**(A)** Nanosecond pulse waveform diagram. **(B)** Microsecond pulse waveform diagram. **(C)** Synergistic pulse waveform diagram.

**Figure 3:**
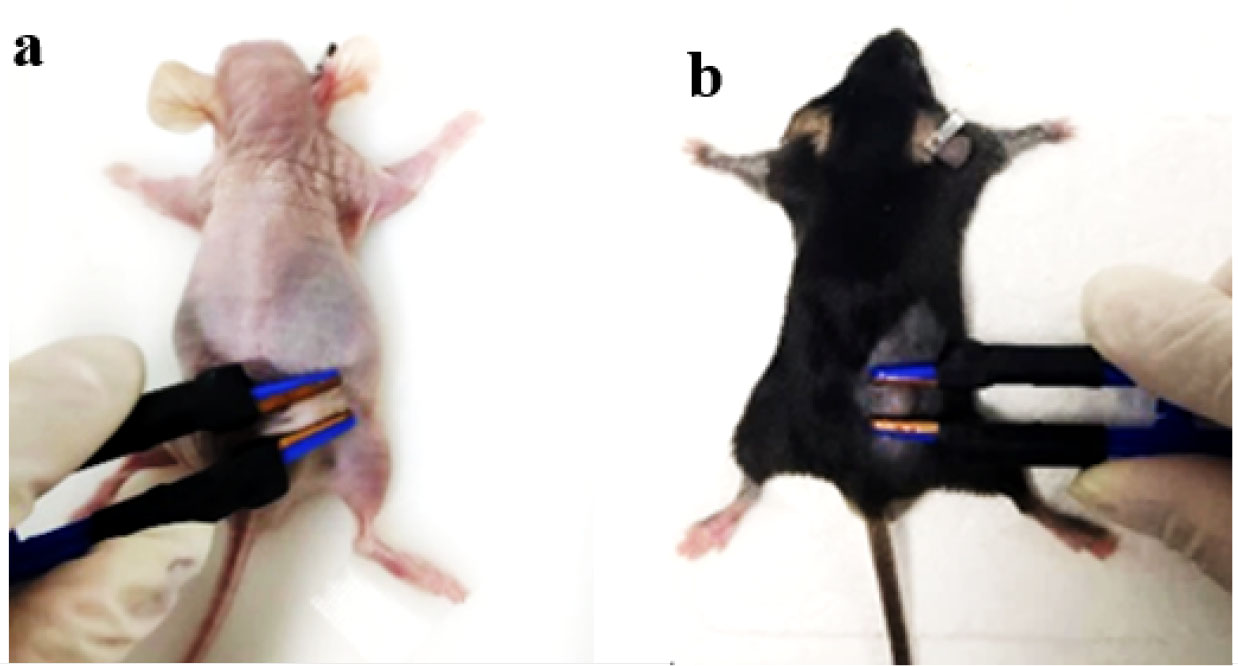
**(A)** Schematic of the nude mouse experiment **(B)** Schematic of the black mouse experiment.

The 20 mice were divided into two groups: a synergistic pulse-treated experimental group and a tumor control group. The experimental group comprised 5 male Balb/c nude mice and 5 male C57BL/6 mice, while the control group included the remaining mice. This study employs a synergistic pulsed electric field composed of nanosecond pulsed electric fields (nsPEFs) and microsecond pulsed electric fields (μsPEFs). A single synergistic pulse unit consists of one nanosecond pulse and one microsecond pulse. When 30 synergistic pulse units are administered per group, 30 nsPEFs and 30 μsPEFs are simultaneously delivered, which is denoted as 30 + 30p. Preliminary experiments have clarified the parametric rules governing the tumor cell-killing effect of the synergistic pulsed electric field, and the pulsed electric field parameters in this study were determined accordingly. This study’s pulsed electric field parameters are shown in **Table 1**. Their waveform is presented in **Figure 2**, with a consistent pulse frequency of 1 Hz and an interval time of 10 μs. (1) Control group: The 10 mice received no synergistic pulse treatment. (2) Experimental group: Each of the 10 mice received 5 treatment cycles, with each cycle consisting of 20 + 20synergistic pulses. Self-made flat plate electrodes (1 cm in length, 4 mm in width) were used for all treatments. After adjusting the electrode spacing (2 mm–5 mm) according to the actual tumor size, the pulse voltage parameters were set to maintain a constant electric field intensity. The specific experimental setups are illustrated in (**Figure 3A**) and (**Figure 3B**), respectively. In this study, the adopted electrode structure was idealized as a parallel-plate capacitor model. Under this model, the electric field strength between the two plates is given by (Equation 2). Given the plate separation distance precisely measured with a vernier caliper, combined with the target electric field strength requirement, the required amplitude of the applied pulse voltage can be calculated accordingly based on this formula, thereby achieving precise control of the electric field strength. Twenty-four hours after treatment, the nude mice were euthanized by intraperitoneal injection of 1% sodium pentobarbital (prepared with physiological saline at an injection dose of 150 mg/kg). After confirmation of death, the tumor tissues were aseptically dissected, and tissue sections were prepared following the pathological section preparation procedure. Four days after treatment, the C57BL/6 mice were euthanized using the same protocol described above. After death was confirmed, the tumor tissues were aseptically excised and processed into pathological sections.

**Table 1:** Experimental pulse parameters.

ns		μs		The number of pulses
Pulse width (ns)	Field strength (kV/cm)	Pulse width (μs)	Field strength (kV/cm)	
500	6	100	1	20 + 20p

(2) $$E\;=\;\frac{U}{L}$$

Among them, *E*, *U*, and *L* are respectively the electric field strength, the voltage between the plates, and the plate separation distance.

#### 2.1.4 Masson staining

Masson staining stains collagen fibers blue and muscle fibers red, respectively. Nude mouse tumor sections were subjected to this staining. These sections were then scanned using a digital pathological section scanner (KF-PRO-005, KFBIO, China). Histopathological changes were analyzed with KF-Viewer and ImageJ software.

#### 2.1.5 Immunofluorescence staining

Immunofluorescence technology was used to detect relevant indicators in nude mouse tumor sections. The primary antibodies included those against carbonic anhydrase IX (CA-IX), hypoxia-inducible factor 1-α (HIF-1α), hyaluronic acid-binding protein (C1QBP), lysyl oxidase (LOX), and fibroblast activation protein 1 (FAP1). Their respective manufacturers and lot numbers are as follows: Affinity (Lot#DF13218 for CA-IX), Cell Signaling Technology (D1S7W Lot#36169 for HIF-1α), Affinity (Lot#DF6675 for C1QBP, Lot#DF13251 for LOX, and Lot#AF5344 for FAP1).

The secondary antibody was Alexa Fluor™ 647 fluorescent dye (Invitrogen, Lot# A-31573), and DAPI fluorescent dye (Biyuntian, Lot#C1002) was used for nuclear labeling. Alexa Fluor™ 647 emits red fluorescence at 668 nm when excited by 633 nm wavelength light. DAPI emits blue fluorescence at 461 nm when excited by 358 nm wavelength ultraviolet light. After staining, the sections were observed and fluorescently imaged using a laser scanning confocal microscope (Zeiss LSM780, Zeiss, Germany). Histopathological changes of the sections were analyzed via ZEN lite2012 software and ImageJ software.

#### 2.1.6 Immunohistochemical staining

Immunohistochemical (IHC) staining was performed on C57BL/6 mouse tumor sections to label immunocytes in tumors and observe their infiltration status. The antibodies used included CD4 rabbit polyclonal antibody, CD8 alpha rabbit polyclonal antibody, NK1.1 Monoclonal Antibody (PK136), and FOXP3 rabbit polyclonal antibody. The CD4 antibody (Servicebio, Lot#GB11064) was used to detect CD4+ T lymphocytes, and the CD8 alpha antibody (Servicebio, Lot#GB11068) for CD8+ T lymphocytes. The NK1.1 antibody (Invitrogen, Lot#WI3392542) was employed to detect NK cells, while the FOXP3 antibody (Servicebio, Lot#GB11093) for regulatory T (Treg) cells. In IHC staining, positive results from the specific binding of antigens to antibodies appear brown. Cell nuclei are stained light blue with hematoxylin dye to enable cell labeling and localization. Stained sections were scanned using a scanner (KF-PRO-005, KFBIO, China). Histopathological changes were analyzed with KF-Viewer and ImageJ software.

### 2.2 Synergistic pulsed electric field combined with adoptively transferred NK cell therapy and *in vivo* imaging

#### 2.2.1 Therapeutic protocol

Specifically, this experiment focused on the intratumoral infiltration of exogenously injected NK cells after synergistic pulse treatment. The experiment was initiated when the tumors grew to approximately 500 mm³.For the experimental group: 2 hours after pulsed electric field treatment, the mice were injected with 200 μL of indocyanine green (ICG)-labeled NK cell suspension via the tail vein. The suspension was prepared with PBS at a cell concentration of 2.5×10^7^ cells/mL.

The control group received no pulsed electric field treatment. Both groups consisted of 5 mice each. The specific methods for animal housing, tumor-bearing mouse model establishment, pre-experiment preparation, experimental platform, and wiring—along with the animal experimental procedures and parameter settings—were consistent with the referenced protocol.

All the above were implemented in accordance with the methods outlined in Section II.A of this paper.

#### 2.2.2 Small animal imaging technology analysis

After the experiment of synergistic pulse-assisted exogenous NK cell injection therapy, a small animal *in vivo* imaging system (IVIS, PerkinElmer, USA) equipped with a gas anesthesia device was used at different time points: before NK cell injection, and at 30 min, 1 h, 2 h, 4 h, 6 h, 8 h, 10 h, and 24 h post-injection. Indocyanine green (ICG) fluorescence was excited at a wavelength of 780 nm (near-infrared region I, NIR-I), with an emission wavelength of 840 nm. This setup was used to observe and image the enrichment of NK cells at the tumor site. At 24 h post-injection, the mice were euthanized. Their hearts, livers, spleens, lungs, kidneys, and tumors were harvested for ex vivo photography. The same imaging system was then used to perform fluorescence imaging of ICG in these different organs. Subsequently, the tumors were dissected to observe the infiltration of NK cells inside the tumors. Data analysis of the imaging results was conducted via Living Image software.

### 2.3 Statistical analysis

All experiments were repeated at least three times, and the experimental data were expressed as mean ± standard deviation (SD). Excel and GraphPad Prism software were used for data analysis. Prior to the analysis, the normality of the data was verified using the Shapiro-Wilk test (W values ranged from 0.92 to 0.98 in all groups, P > 0.05), and the homogeneity of variance was confirmed via the Levene test (F values ranged from 1.03 to 1.87 in all groups, P > 0.05). After confirming that the applicable prerequisites for one-way analysis of variance (one-way ANOVA) were satisfied, this method was employed to test the significance of differences among groups. The significance levels were defined as follows: *** indicates P < 0.001, ** indicates 0.001 < P < 0.01, and * indicates 0.01 < P < 0.05.

## 3 Results and analysis

### 3.1 Synergistic pulse improves the tumor physicochemical microenvironment

**Figure 4:**
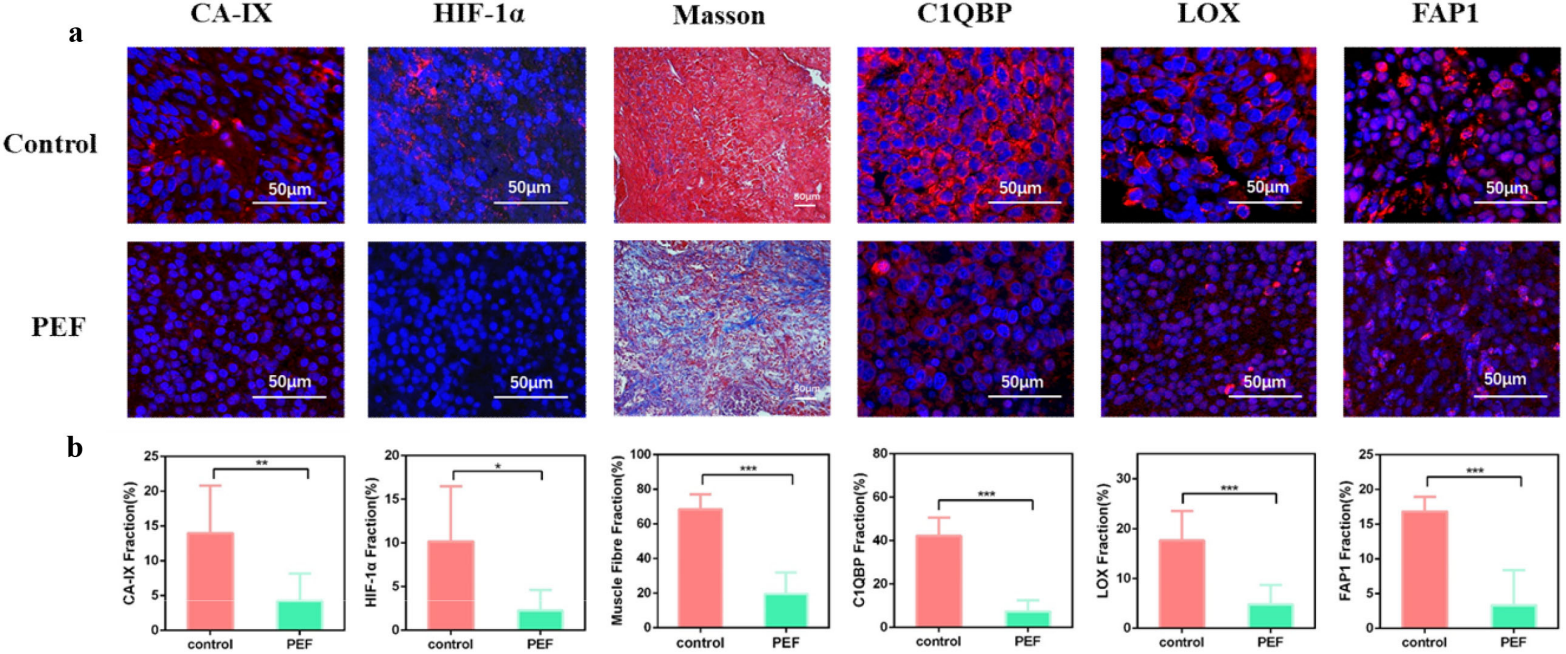
**(A)** Fluorescence staining images of CA-IX, HIF-1α, muscle fiber content, C1QBP, LOX, and FAP before and after treatment. **(B)** Statistical graphs of CA-IX, HIF-1α, muscle fiber content, C1QBP, LOX, and FAP before and after treatment. * indicates P<0.05, ** indicates 0.001<P<0.01, *** indicates P<0.001, representing statistical significance between the control group and PEF group, determined by one-way ANOVA.

Carbonic Anhydrase IX (CA-IX) Antibody (Affinity, Lot#DF13218) and HIF-1α (D1S7W) XP^®^ Rabbit mAb (Cell Signaling Technology, Lot#36169) were used to assess tumor cell hypoxia. As shown in (**Figures 4A, B**), CA-IX and HIF-1α fluorescence intensities were significantly lower in the synergistic pulse group than in the non-electric pulse control group. The positive expression rates of CA-IX were 14.016 ± 6.800% in the control group and 4.270 ± 3.904% in the synergistic pulse-treated group, respectively. For HIF-1α, the positive expression rates were 10.169 ± 6.321% (control group) and 2.263 ± 2.363% (synergistic pulse-treated group). These results indicate that synergistic pulse effectively ameliorated the tumor hypoxic microenvironment.

Hyaluronic Acid Binding Protein (C1QBP) Antibody (Affinity, Lot#DF6675) binds ECM hyaluronic acid, mediates cell-matrix adhesion, and enhances ECM hydrostatic pressure. Lysyl Oxidase (LOX) Antibody (Affinity, Lot#DF13251) is cell-secreted, induces collagen and elastin cross-linking and fibrosis, and promotes ECM sclerosis. FAP (Fibroblast Activation Protein, FAP-α) is expressed on the surface of cancer-associated fibroblasts (CAFs). When activated by interaction with cancer cells, it produces and secretes structural proteins and adhesion proteins in the ECM, further remodeling the ECM and increasing its stiffness. High expression of C1QBP, LOX, and FAP in tumors can further inhibit immune cell infiltration and promote tumor growth, migration, and invasion. Combined with Masson staining (collagen fibers blue, muscle fibers red) and immunofluorescence (**Figures 4A, B**), collagen fibers—the main tumor stiffness indicator—showed a loose network in the synergistic pulse group. Meanwhile, LOX (an enzyme mediating collagen cross-linking) decreased from 17.632 ± 5.912% (control group) to 4.763 ± 3.923% (synergistic pulse group). The “muscle fibers” specifically refer to α-SMA-positive myofibroblasts in tumors and their secreted actin fibers (not host muscle tissue), and their content decreased from 68.460 ± 8.626% (control group) to 19.479 ± 12.591% (synergistic pulse group). This downward trend was consistent with that of FAP (a marker of CAFs), whose expression dropped from 16.850 ± 2.122% in the control group to 3.343 ± 5.062% in the synergistic pulse group—serving as auxiliary evidence for collagen regulation. Additionally, the expression of C1QBP decreased from 42.246 ± 8.222% (control group) to 7.366 ± 5.159% (synergistic pulse group). Thus, synergistic pulse treatment effectively controlled tumor stiffness and density.

**Figure 5:**
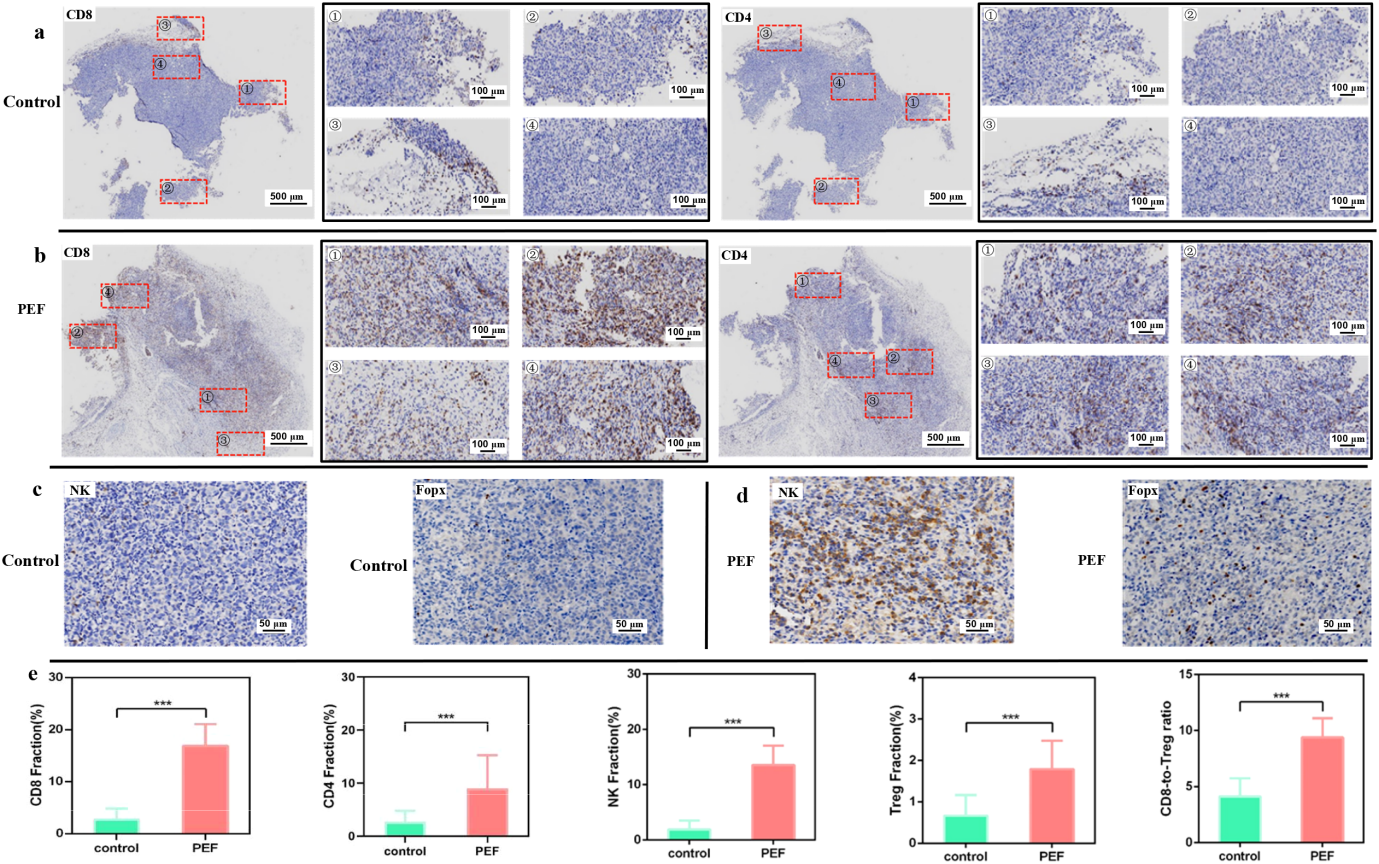
**(A)** IHC staining of CD8^+^ T cells and CD4^+^ T cells in the control group. **(B)** IHC staining of CD8^+^ T cells and CD4^+^ T cells in the experimental group. **(C)** IHC staining of NK cells and regulatory T cells (Treg cells) in the control group. **(D)** IHC staining of NK cells and rTreg cells in the experimental group. **(E)** Statistical comparison of intratumoral infiltration levels of CD8^+^ T cells, CD4^+^ T cells, NK cells and Treg cells between the control and experimental groups. *** indicates P<0.001, representing statistical significance between the control group and PEF group, determined by one-way ANOVA.

**Figure 5** shows the distribution characteristics of CD8^+^ T cells, CD4^+^ T cells, NK cells and Treg cells in tissue sections of the experimental group and the control group under pulsed electric field intervention. Among them, the stained sections of the four immune cell types in the control group were obtained from different consecutive sections of the same tumor tissue, and the stained sections of the four immune cell types in the experimental group were obtained from different consecutive sections of another corresponding tumor tissue. CD8^+^ T lymphocytes are cytotoxic killer T cells, which can specifically kill tumor target cells by secreting perforin and granzyme, releasing TNF-α and IFN-γ, and via the FasL pathway. CD4^+^ T lymphocytes are helper T cells, mainly including four subsets: TH1, TH2, TH17 and Treg cells, which can directly or indirectly regulate the activity of other immune cells through the synthesis and secretion of related cytokines. As shown in (**Figure 5A**), tumor cells in the control group were densely arranged, and CD8^+^ T cells and CD4^+^ T cells could hardly infiltrate into the tumor parenchyma, but only distributed in small amounts at the edge of the tumor tissue. In contrast, after synergistic pulsed electric field treatment (**Figure 5B**), the tumor tissue became significantly looser, the number of CD8^+^ T cells and CD4^+^ T cells at the tumor edge increased, and a large number of these cells began to infiltrate into the tumor interior. The distribution characteristics of NK cells and Treg cells showed similar trends in (**Figures 5C, D**). Quantitative analysis results in (**Figure 5E**) showed that the positive rates of CD8^+^ T cells and CD4^+^ T cells in the tumor tissue of the control group were at low levels (2.749 ± 2.059% and 2.684 ± 2.085%, respectively). After synergistic pulse treatment, the positive rates of both were significantly increased and widely distributed (16.878 ± 4.177% and 9.014 ± 6.241%, respectively). Meanwhile, the positive rate of NK cells in tumors was significantly up-regulated from 1.922 ± 1.602% in the control group to 13.561 ± 3.479% after synergistic pulse treatment. Treg cells can inhibit the activation of CD4^+^ T cells and CD8^+^ T cells, thereby weakening the antitumor immune response in the tumor microenvironment and promoting tumor growth and progression. In this study, the infiltration level of immunosuppressive Treg cells in tumors was also significantly increased from 0.668 ± 0.496% in the control group to 1.797 ± 0.684% in the synergistic pulse group. However, the CD8^+^ T/Treg ratio was still significantly increased from 4.116 ± 1.630% in the control group to 9.393 ± 1.697% after synergistic pulse treatment.

These findings suggest that synergistic pulse stimulates the body’s intrinsic immunity, induces local tumor pro-inflammatory responses, and recruits and promotes immune cell infiltration into tumors. It is expected to further attack and eliminate residual tumor cells after synergistic pulse ablation, and prevent tumor recurrence.

### 3.2 Synergistic pulse promotes intratumoral infiltration of exogenously injected NK cells

**Figure 6:**
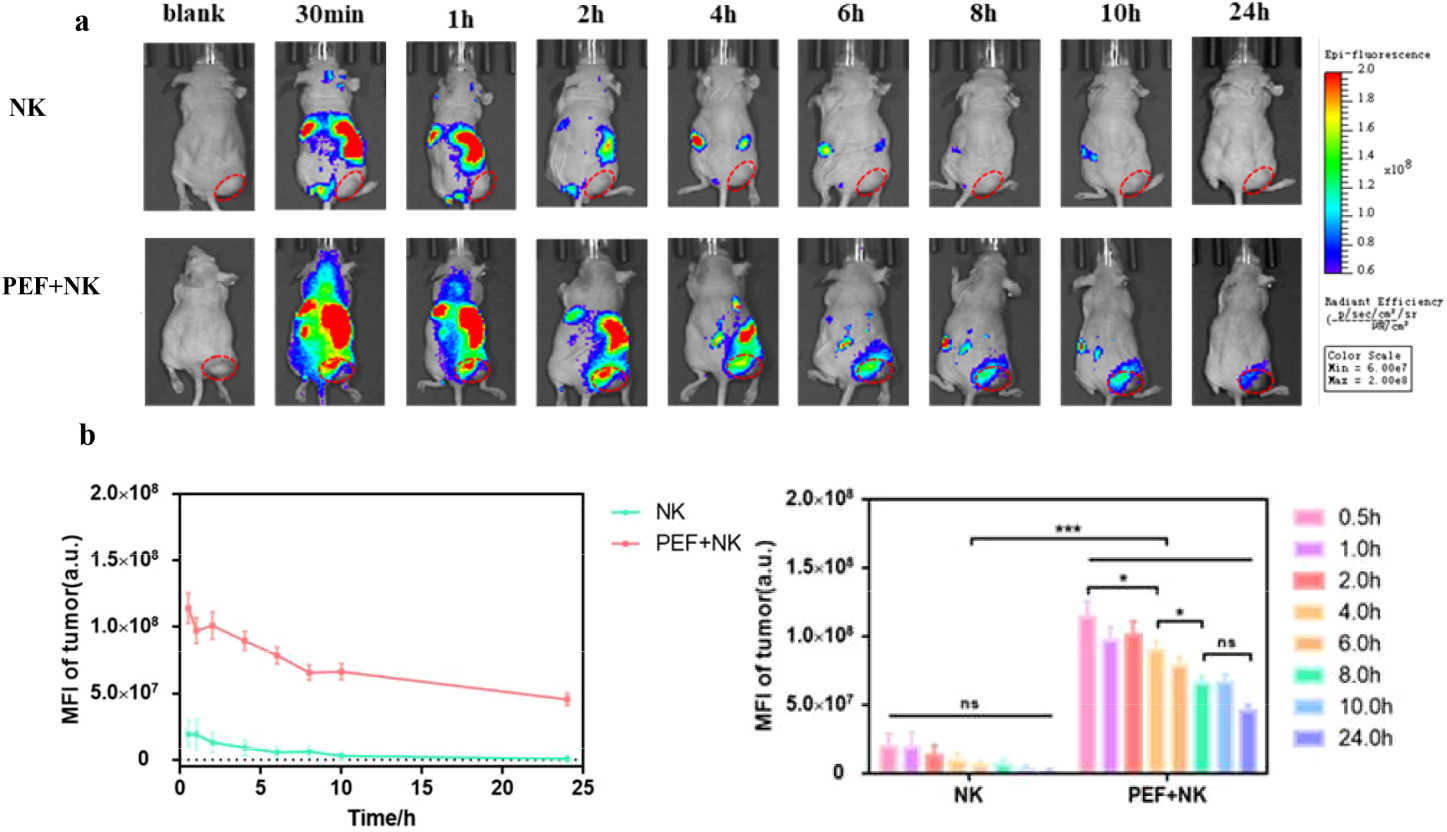
**(A)** Time-dependent ICG fluorescence imaging results of tumor-bearing mice *in vivo* **(B)** Curves of changes in the average ICG fluorescence intensity of NK cells at the in vivo tumor site before and after treatment. * indicates P<0.05, *** indicates P<0.001, representing statistical significance between the NK group and PEF+NK group, determined by one-way ANOVA. ns is the abbreviation of "not significant", indicating no statistically significant difference between the compared groups (P>0.05).

**Figure 7:**
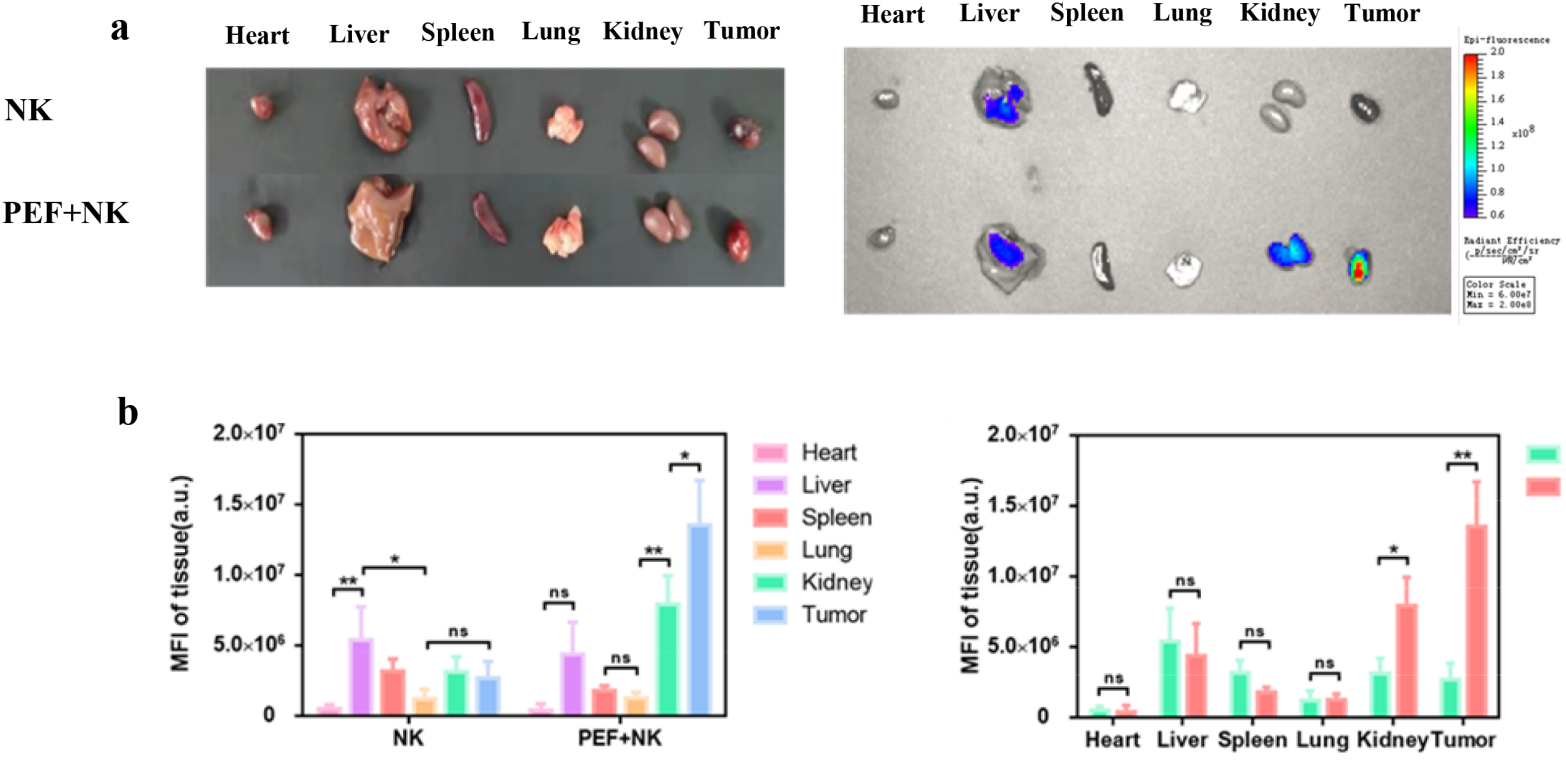
**(A)** Statistical graph of the average ICG fluorescence intensity of NK cells at the in vivo tumor site before and after treatment **(B)** Ex vivo imaging results of major organs and tumors, and statistical graph of their average ICG fluorescence intensity. * indicates P<0.05, ** indicates 0.001<P<0.01, representing statistical significance between the NK group and PEF+NK group, determined by one-way ANOVA. ns is the abbreviation of "not significant", indicating no statistically significant difference between the compared groups (P>0.05).

**Figure 8:**
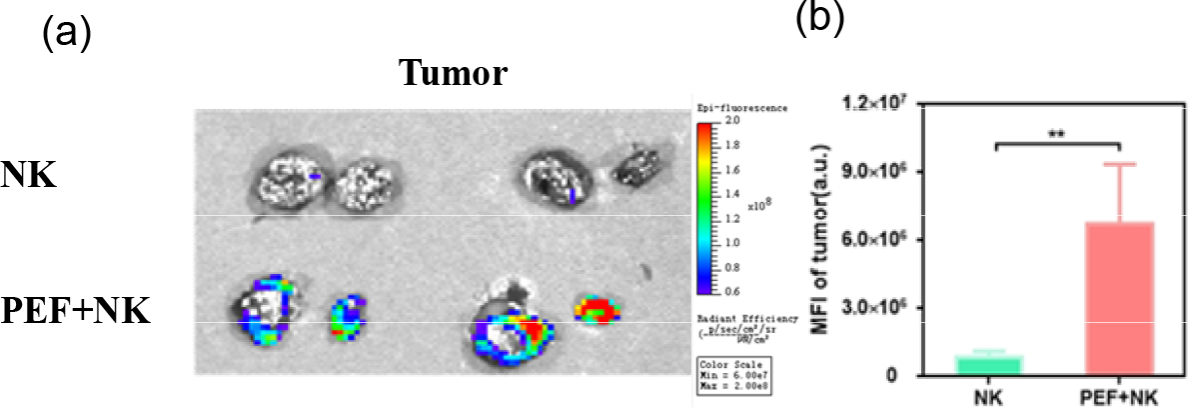
**(A)** Ex vivo imaging results of dissected tumors **(B)** Statistical graph of their average ICG fluorescence intensity. ** indicates 0.001<P<0.01, representing statistical significance between the NK group and PEF+NK group, determined by one-way ANOVA.

(**Figure 6A**) shows the time-dependent changes in *in vivo* ICG fluorescence imaging of tumor-bearing mice. The control group received no synergistic pulsed electric field treatment and only NK cell injection, while the experimental group was injected with NK cells after synergistic pulsed electric field treatment. Statistical analysis of the mean ICG fluorescence intensity at the tumor site in *in vivo* imaging yielded the results presented in (**Figure 6B**). It can be observed that ICG fluorescence intensity decreased over time after NK cell injection. Moreover, tumor-site ICG fluorescence intensity was low in the control group, while significantly higher in the synergistic pulse-treated group (***). **Figure 7** displays the ex vivo imaging results and statistical graphs of mean ICG fluorescence intensity for major organs (heart, liver, spleen, lungs and kidneys) and tumors of tumor-bearing mice at 24 hours post *in vivo* imaging. There was a significant difference in ICG fluorescence intensity in tumors between the control group and the experimental group (**). Additionally, tumor-site ICG fluorescence intensity was much higher in the synergistic pulse-treated group than in other major organs (heart, liver, spleen, lungs, kidneys). This indicates that NK cells were mainly concentrated at the tumor site after synergistic pulsed electric field treatment. Due to *in vivo* metabolic processes, the ICG fluorescence intensity in metabolic organs (liver and kidneys) was slightly higher. In contrast, the fluorescence intensity in the heart was extremely low, suggesting that this method had no cardiac toxicity, and ICG fluorescent molecules and NK cell debris did not enter the heart. Dissection of tumor tissues revealed the ex vivo imaging results and statistical graphs of mean ICG fluorescence intensity shown in **Figure 8**. As indicated in the figure, ICG fluorescence was present inside the tumors, with intensity significantly higher than that in the control group (**). This suggests that synergistic pulsed electric field not only enabled NK cell enrichment at the tumor site but also promoted their penetration and infiltration into the tumor interior.

## 4 Discussion

In this study, tumor-bearing models constructed using male Balb/c nude mice and male C57BL/6 mice were employed as research carriers. This study represents the first multi-faceted analysis of the tumor microenvironment (TME) remodeling effects induced by a novel synergistic pulse protocol combining high-voltage nanosecond and low-voltage microsecond pulses. Furthermore, it elucidates the molecular mechanisms and biological outcomes underlying the enhanced efficacy of externally infused NK cell therapy facilitated by this approach. Its core innovative value lies in filling the research gap in the field of TME regulation by synergistic pulses. From the mechanistic analysis of experimental results, the regulation of TME by synergistic pulses exhibits dual synergistic characteristics: optimization of the physical microenvironment and activation of the immune microenvironment. Moreover, it demonstrates significant targeted enhancement effects and favorable biosafety in combination with cellular immunotherapy.

At the tumor physical microenvironment level, synergistic pulse treatment significantly reduced the positive expression rates of CA-IX and HIF-1α in tumor tissues. This indicates effective alleviation of tumor hypoxia, consistent with traditional irreversible electroporation (IRE)’s trend in improving microenvironmental hypoxia. In terms of ECM remodeling, Masson and immunofluorescence results showed significant reduction in tumor muscle fiber content after synergistic pulse treatment. Collagen fibers with a loose network structure were selectively retained. Meanwhile, the expression levels of C1QBP, LOX, and FAP were significantly downregulated. This change breaks the physical barrier of the TME at the molecular level. On the one hand, reducing C1QBP expression weakens the adhesion between tumor cells and the matrix, lowering the hydrostatic pressure of the ECM. On the other hand, inhibiting LOX-mediated collagen cross-linking and FAP-regulated CAF activity reduces ECM structural protein synthesis and deposition. Ultimately, the originally dense and rigid ECM becomes loose and softened, creating a structural basis for immune cell infiltration and the delivery of exogenous therapeutic agents. This further indirectly inhibits the proliferation, invasion, and migration of tumor cells.

At the level of immune microenvironment activation, synergistic pulses promote cytotoxic immune cell infiltration.

This effect can be explained by a dual mechanism of “physical channel construction + immune signal activation”. Physically, the loosening of the ECM provides structural channels for CD8^+^T cells, CD4^+^T cells and NK cells to penetrate the tumor parenchyma. This effectively addresses the “infiltration barrier” issue in the control group, where immune cells only remained at the tumor edge. Immunologically, synergistic pulse-mediated tumor cell damage releases damage-associated molecular patterns (DAMPs). These molecules in turn trigger local pro-inflammatory responses and actively recruit endogenous immune cells to migrate to the tumor site. Immunohistochemical results showed that the positive expression rates of CD8^+^T cells, CD4^+^T cells and NK cells in tumor tissues were significantly increased after synergistic pulse treatment. As core cytotoxic immune cells, CD8^+^T cells secrete perforin, granzyme and cytokines to specifically kill tumor target cells. CD4^+^T cells regulate the activity of other immune cells by secreting cytokines, collectively enhancing the anti-tumor immune response. Notably, the number of immunosuppressive regulatory T cells (Treg) slightly increased after synergistic pulse treatment, while the proliferation and activation of CD8^+^T cells were more significant. This led to a substantial increase in the CD8/Treg ratio, a key indicator for evaluating the anti-tumor immune dominance of the TME. Its elevation provides an important immune basis for eliminating residual tumor cells after ablation and inhibiting tumor recurrence.

In the study of synergistic pulse-assisted exogenously injected NK cell therapy, *in vivo* small animal imaging was performed. The results showed that the ICG fluorescence intensity at the tumor site in the experimental group was significantly higher than that in the control group. After 24 hours, tumor tissue fluorescence accumulation was much higher than that in major organs (heart, liver, spleen, lungs, kidneys), with extremely low intensity in the heart. This confirms the targeting and biosafety of the combined regimen. The core reason for the poor therapeutic effect of NK cell therapy in the control group lies in the TME’s multiple inhibitory effects. Firstly, NK cells are relatively large and cannot accumulate in solid tumors via the EPR effect of tumor blood vessels like nanoparticles. Secondly, the dense ECM spatially isolates NK cells from tumor cells. Thirdly, tumor hypoxia downregulates membrane MICA expression by inducing HIF-1α accumulation in tumor cells, reducing their sensitivity to NK cell-mediated lysis. Meanwhile, HIF-1α can inhibit the expression of NK cell activation receptors (such as NKp46 and NKp30), impairing their killing function. Synergistic pulses specifically address the above issues by remodeling the TME. The loosening of the ECM eliminates the spatial barrier, and the alleviation of hypoxia reverses the immune resistance of tumor cells and the functional inhibition of NK cells. At the same time, local pro-inflammatory responses further recruit exogenously injected NK cells. Ultimately, this enables efficient NK cell accumulation and intratumoral penetration at the tumor site, markedly enhancing cellular immunotherapy efficacy. Meanwhile, this experiment emphasizes that synergistic pulses can construct a more “permissive” tumor microenvironment with alleviated hypoxia, providing a key physical and chemical basis for immune cell infiltration and functional exertion. On the other hand, it clarifies that synergistic pulses act as an immunotherapy sensitizing agent. They are expected to overcome the core clinical challenges of poor immune cell targeting and weak penetration in solid tumor treatment.

This study has only verified the effect of synergistic pulses through the hepatocellular carcinoma model, and its generalizability still needs to be further confirmed through multiple models in subsequent research. Furthermore, the current study mainly focuses on verifying the overall efficacy of the combined protocol, and its direct comparative advantages over each monotherapy will be a key focus of subsequent research. For example, we will further optimize the tumor model to enhance clinical relevance and conduct verification in combination with more types of tumor models, so as to comprehensively evaluate the generalizability and clinical translation potential of synergistic pulses.

## 5 Conclusion

For the first time, we detected TME physicochemical indicators (density, stiffness, hypoxia) and various immune cell subsets post synergistic pulse treatment. We found that synergistic pulsed electric fields downregulated CA-IX and HIF-1α, while downregulating muscle fiber content, C1QBP, LOX, and FAP in the TME. These results reveal that synergistic pulses modulate tumor stroma to soften and loosen it, reduce tumor density and stiffness, and alleviate TME hypoxia and immunosuppression. After synergistic pulse treatment, tumor tissues showed high CD8, CD4, and NK1.1 expression. This indicates that synergistic pulses elicit the body’s innate immune response and promote intratumoral infiltration of immune cells (CD8+ T cells, CD4+ T cells, NK cells). Via small animal *in vivo* imaging, we further confirmed that synergistic pulses enhance exogenously injected NK cell accumulation and intratumoral penetration at the tumor site. Collectively, this work demonstrates that synergistic pulses improve the physical and immune microenvironment of heterogeneous tumors post ablation. This can provide mechanistic support for the targeted delivery of NK cells by improving the TME. Meanwhile, this study provides preliminary evidence for the subsequent exploration of the potential value of this technology in tumor recurrence and metastasis prevention.

## Data Availability

The raw data supporting the conclusions of this article will be made available by the authors, without undue reservation.
